# Oral Medicine: a retrospective analysis of patient profiles, diagnoses, and referral patterns in Mexico City

**DOI:** 10.4317/medoral.27481

**Published:** 2025-08-16

**Authors:** Alexia Mariana Figueroa-Ramos, Gabriela Anaya-Saavedra, María Esther Irigoyen-Camacho, Adalberto Mosqueda-Taylor

**Affiliations:** 1 Postgraduate Program in Oral Pathology and Medicine, Health Care Department, Metropolitan Autonomous University, Mexico City, Mexico

## Abstract

**Background:**

Oral Medicine (OM) is a dental specialty dedicated to diagnosing and non-surgical managing of oral diseases, often in medically compromised patients. In Mexico, no prior studies have examined the scope of the clinical practice of this specialty; thus, this study aims to examine the characteristics of patients treated at three different reference centers of OM in Mexico City, focusing on their diagnoses, sources of referral, and treatments.

**Material and Methods:**

This retrospective, observational and descriptive study reviewed 1,270 clinical records from three specialized OM centers between 2015 and 2022: a university clinic, an intrahospital service and a private OM practice. Demographic, clinical and therapeutic data were collected and analyzed using JMP Pro 16, with a significance level of *p* <0.05.

**Results:**

Most patients were women (71.6%), primarily in their sixth and seventh decades of life. Comorbidities were present in 74.7% of cases, and 63.2% of patients were on medications. In public institutions, the most frequent reason for consultation were tumors, while in the private clinic, it was burning mouth. Dentists were the main referrers (49%), yet only 24.2% of referrals included a diagnosis, of which 60% were accurate. The most prevalent oral conditions were non-odontogenic infections (26.4%), ulcerative and vesiculobullous lesions (15.2%), and oral potentially malignant disorders (14.8%). Pharmacologic treatment was provided to 71% of patients, while management of 29.9% included consultation with other specialists.

**Conclusions:**

This study highlights significant differences in patient profiles, diagnosis, and referral accuracy between the public and private services of OM. The low diagnostic precision in medical and dental referrals underscores the need to strengthen OM education. Additionally, the wide spectrum of oral and maxillofacial disorders and their treatments emphasize the importance of multidisciplinary management and integration with other medical specialties. These findings support the need to evaluate educational programs, optimize referral pathways, and improve the quality of OM care in Mexico.

** Key words:**Oral medicine, stomatology, oral diseases, epidemiology.

## Introduction

Oral Medicine (OM) is a relatively recent dental specialty (1) concerned with the diagnosis and non-surgical treatment of patients with chronic, recurrent and medically related disorders of the oral and maxillofacial region (2). This specialty serves as a critical link between medical and dental care (3), since it does not only focus on treating complex oral pathologies but also collaborates with various medical disciplines to ensure a comprehensive approach for patients with diverse oral and maxillofacial disorders.

OM is practiced in multiple countries and across different healthcare settings, including clinics, hospitals, dental schools and private services (3). Its clinical relevance is based on the fact that OM specialists frequently manage patients who have undergone unsuccessful treatment by healthcare professionals (4-8), lacking the necessary knowledge, experience, or skills to accurately diagnose and treat their conditions (4).

The recognition of OM as a formal dental specialty varies worldwide. In some countries, it is officially recognized as a distinct specialty (9-11), while in others, it remains as an area of study within the broader dental curriculum (10). Internationally, professional training in OM exhibits significant variation (12). Currently, at least 22 countries offer formal training OM programs (1) structured independently or in conjunction with other dental specialties, and these range from two to six years, depending on the educational model of each country. Furthermore, they may be university or hospital-based, influencing graduate’s theoretical and practical focus. However, there remains a lack of standardization in curriculum contents (12), which is possibly explained by historical, cultural and socioeconomic differences among regions.

Considering that no previous studies have comprehensively analyzed the clinical practice of OM in Mexico, the main objectives of this study were to characterize the profile of patients treated at three OM reference centers -public and private- in Mexico City, to identify the most common diagnoses and therapeutic interventions required, and to compare these findings with international reports on OM practice.

## Material and Methods

This retrospective, observational, and descriptive study analyzed the clinical records of patients treated at three OM reference centers in Mexico City between January 2015 and December 2022. The participating centers included a public university clinic, a specialized OM clinic affiliated with a Dermatology Department of a large public general hospital, and a private specialized OM practice.

Clinical records were included if patients had attended at least one consultation in any of the three services during the study period. Records with incomplete or inconsistent data were excluded. The collected data encompassed demographic (sex, age, place of birth and residence) and clinical variables, including comorbidities, medication use, source of referral, the reason for consultation, duration of the condition, relevant medical history, diagnostic procedures, therapeutic interventions implemented, the total number of consultations, and referrals to other medical services.

Systemic diseases were classified according to the International Classification of Diseases for Mortality and Morbidity Statistics (ICD-11, version 01/2023). Prescribed medications were categorized using the 22nd WHO Model List of Essential Medicines (2021), supplemented with additional drugs not included. In cases where patients had multiple diagnoses, all identified conditions were documented.

Data processing and statistical analysis were conducted using the JMP Pro (version 16) software. Frequencies were calculated for categorical variables, while medians and interquartile ranges were determined for continuous variables. Bivariate analysis of nominal variables was performed using the Chi-square test, while ordinal variables were analyzed using the Kruskal-Wallis’s test. A statistical significance level of *p* ≤ 0.05 was established.

## Results

A total of 1,270 clinical records were analyzed from the three OM referral centers: 310 from the university clinic, 306 from the hospital clinic, and 654 from the private clinic. As shown in Table 1, 71.6% were women, predominating in their sixth and seventh decades of life. The median age varied across centers, being lower at the university and higher at the private practice. Patients under 20 years old represented only 6% of the total sample. Most participants were born and residing in Mexico City.

The educational levels of the patients varied significantly between centers: the university and private clinics had a higher proportion of individuals with higher education and postgraduate degrees, whereas patients at the hospital clinic predominantly had basic or middle-level education (elementary and high school). Regarding occupation, housework was the most common activity overall; even so, specific trends were observed across centers: the university clinic had a higher proportion of students, the hospital clinic had more employees, and the private clinic had significant proportion of professionals.

A high prevalence of systemic diseases (74.7%) was observed among participants, 63.2% of whom took at least one medication at the time of their first consultation. The most used pharmaceutical drug categories were antihypertensives, proton pump inhibitors, hormone replacement therapies, hypoglycemic agents, antidepressants, and lipid-lowering agents. The median number of consultations was highest at the hospital clinic (4 consultations per patient), with a maximum of 19 consultations per year and 85 in total.

Before seeking OM care, 46.9% of patients consulted other healthcare professionals, some having visited up to six different specialists. Most referrals came from dentists (49.2%), particularly maxillofacial surgeons, followed by medical specialists (41.7%), mainly dermatologists. The distribution of referrals varied across centers: the hospital clinic received more medical referrals (77.4%), the university clinic had mainly dental referrals (83%), and the private clinic had a more balanced distribution (51.2% from dentists and 35.8% from medical doctors). Only 24.2% of referrals included a diagnosis, with an accuracy rate of 60%.

As shown in Table 2, the main reasons for consultation differed between centers: in the university and hospital clinics the primary concern was suspicion of tumors, while in the private clinic, the most frequent reason was burning mouth. The median duration of symptoms before being referred to an OM service was 7 months.

The most common diagnostic procedures (Table 3) included biopsies (29.1%), blood tests (27.2%) and imaging studies (10.4%). The hospital clinic conducted salivary function tests (sialometry and glandular function test) and interdisciplinary consultations more frequently, while at the private practice the use of fluorescent light test (Identafi ®) for evaluating oral potentially malignant disorders was more commonly used.

The most frequent pathological conditions recorded were non-odontogenic infections (26.4%, mainly candidiasis and HPV-lesions), miscellaneous benign oral conditions (20.4%, mainly geographic tongue, frictional keratosis and vascular malformation), ulcerative and vesiculobullous lesions (15.2%, including traumatic ulcer, recurrent aphthous stomatitis and pemphigus vulgaris), oral potentially malignant disorders (14.8%, primarily oral lichenoid disease), and benign reactive and tumoral lesions (11.9%, mainly fibrous hyperplasia and pyogenic granuloma) (Table 4). Among the 76 patients under 20 years of age, the most common oral findings were reactive lesions and benign neoplasms (*n*=20), non-neoplastic salivary gland disorders (*n*=12) (mainly mucocele) and other benign conditions (*n*=10), including geographic tongue and biting habits.

Therapeutic interventions involved prescription of medications (71%), recommendation of therapeutic substances (39.9%), and self-care instructions (19.1%). The prescription of medications and therapeutic substances was similar both in private and hospital centers, while clinical interventions (surgical treatments, elimination of irritating local factors, prophylaxis, tooth extraction, and prosthetic rehabilitation) were more frequently performed at the university clinic (Table 3). The most prescribed drugs included corticosteroids (42.6%), antifungals (39.3%), mucosal surface protectors and combined preparations (19.5%), sialagogues (11.0%), anti-inflammatory drugs and analgesics (10.5%), immunomodulators/immunosuppressants (9.1%), anxiolytics, antidepressants and antipsychotics (5.5%), antibiotics (5.3%), antihistamines (5.0%), anticonvulsants (1.1%), and other drugs (healing promoters, muscle relaxants, antivirals, etc.). Among the complementary therapeutic substances, the most commonly recommended were oral hygiene products (15.2%), nutritional supplements (12.9%), lip moisturizers (10.6%) and saliva substitutes (10.3%).

A significant proportion of patients (29.9%) were referred to other medical or dental services for additional treatments, whether related or unrelated to their reason for OM consultation.

## Discussion

The present study provides an initial approach to the epidemiological and clinical profile of patients treated in specialized OM services in Mexico City, highlighting distinctive patterns between the public and private sectors. NoTable outcomes include the predominance of female patients in their fifth to seventh decades of life, the high prevalence of comorbidities among individuals seeking OM care, variations in referral patterns, and the low diagnostic accuracy among referring professionals. These findings underscore the need for improved training of primary care providers to enhance the recognition and management of oral disorders.

The predominance of female patients in their fifth to seventh decades of life aligns with previous studies (5,8,13-16). This trend may be attributed to the higher prevalence of immunological oral diseases in women and differences in healthcare-seeking behaviors between the sexes (6). Conversely, patients under 20 represented the smallest age group, a pattern also observed in previous reports (5,14). This could be explained by several factors, including the lower incidence of complex oral pathologies in younger individuals, the effective management of common oral disorders by primary care providers, and the frequently acute and self-limiting nature of most conditions. However, this does not diminish the importance of studying oral and maxillofacial conditions in pediatric and adolescent populations to better define OM´s role, scope and limitations in their care, as well as the need to detect early some serious and potentially fatal disorders that may occur with higher frequency during childhood.

The geographic concentration of patients from Mexico City in this study, with only a small proportion coming from other states, may reflect the growing availability of OM and oral pathology specialists nationwide, potentially reducing the need for referrals to the capital. Longitudinal patient follow-ups revealed sustained consultation over extended periods, consistent with the chronic nature of many conditions treated in OM (6,8,17).

A significant finding, consistent with previous reports (5,14), is the high prevalence of diverse systemic comorbidities and the widespread use of multiple medications and therapeutic substances among OM patients. As others had reported (7,13), the most common conditions found were cardiovascular, endocrine, rheumatological, digestive and psychiatric disorders. Although these diseases do not necessarily have a direct causal association with specific oral conditions, the pharmacological agents used to manage them are often linked to oral manifestations such as lichenoid disease and hyposalivation. These findings highlight the importance of characterizing the epidemiological profile of comorbidities in OM patients to optimize comprehensive care, enhance interdisciplinary collaboration, and improve clinical outcomes in hospital settings.

Consultation patterns varied between centers. In private practice, the leading reason for consultation were burning and ulcerative disorders, whereas in the university and hospital settings, it was tumors. These findings contrast with studies from developed countries (7,14,15), where white or red lesions are the most common reason for OM consultation. This disparity may be influenced by several factors, including differences in healthcare professionals’ training in oral lesion identification, variations in public health awareness, or region-specific incidence rates linked to distinct environmental, behavioral and cultural factors.

The median duration of symptoms before an OM consultation was seven months, considerably shorter than the 16.8 months reported in the U.S.A. (7). Referral patterns differed across centers: in the university clinic dental referrals were predominant, likely due to greater recognition of OM services within the local dental community. In the hospital setting, medical referrals were more common, consistent with previous studies showing that university clinics receive more referrals from dentists, and hospitals primarily receive referrals from physicians (6,8,15). At private practice, the proportion of medical and dental referrals was more balanced. Among dental specialists, maxillofacial surgery was the leading source of referrals, whereas, among medical specialists, dermatology predominated, reflecting the close clinical and pathological overlap between these fields and OM.

A noteworthy finding, consistent with previous reports (8,15,18,19), is the low frequency of referrals accompanied by a diagnostic hypothesis, mainly due to the absence of OM-specific contents in the curricula of most medical schools worldwide (20-23). This, combined with the high number of unnecessary diagnostic and therapeutic tests identified, underscores the urgent need to strengthen continuing education programs for healthcare professionals. The consequences of these knowledge deficiencies are significant, as these contribute to delays in diagnosis and treatment, increased healthcare costs, and unnecessary patient burden.

The diagnostic procedures documented in this report were diverse and coincided with previous research (5,7,8,16). University and hospital centers showed a higher frequency of blood and imaging tests, as well as a greater volume of biopsies compared to private practice. These differences suggest the need for evidence-based diagnostic protocols to prevent the overprescription of tests, that not only increases patients’ cost, but can delay the diagnosis and treatment. Selection of diagnostic studies should be guided by a thorough clinical history and physical examination, ensuring that only essential tests are ordered, thereby optimizing diagnostic efficiency and resource allocation.

The results of this study confirm that OM encompasses a broad and diverse spectrum of pathological disorders affecting the buccal and maxillofacial complex. While most studies identify immune-mediated disorders as among the most commonly diagnosed and treated conditions in OM (5,7,8,13-15,24), our findings indicate that non-odontogenic infections accounted for a quarter of cases, with oral candidiasis being particularly prevalent. This high frequency could be attributed to the large number of dermatology patients undergoing steroid therapy for immune-mediated conditions such as pemphigus vulgaris, pemphigoid, and lichenoid diseases, as well as a high percentage of non-controlled diabetic patients and other causes of immunosupression.

In the pediatric population, our findings suggest that traumatic, reactive, infectious, and ulcerative lesions, as well as normal variants, are the most frequent conditions, which is in agreement with the available literature (25,26). However, significant geographical variability exists in the global distribution of oral diseases in children. In regions of Africa and Asia, neoplasms, potentially malignant disorders, and severe infections are more prevalent (26,27), whereas in America and Europe, reactive lesions and benign tumors are more commonly reported (26).

The profile of therapeutic interventions observed in our study closely resembles those reported in the United States, Canada, Australia, New Zealand, and China (5,14,16), where pharmacological treatments predominated, along with the use of other therapeutic substances and self-care recommendations. The variety of prescribed medications in OM contrasts significantly with other dental specialties, where analgesics and antibiotics are the predominant pharmacological agents prescribed. This disparity underscores the complexity of OM, which requires specialized knowledge in general and specific pharmacology for managing diseases affecting the oral, maxillofacial and adjacent anatomical structures, as well as neurofunctional disorders.

OM specialists must consider potential drug interactions, given that patients are mainly polymedicated. Beyond pharmacological management, other therapeutical interventions included surgical procedures, dental treatments, dental appliances, elimination of traumatic factors, physiotherapy, and minor oral surgery, reaffirming the broad therapeutic scope of OM. Contrary to practices in other countries, where the dental management of systemically compromised patients is overseen by Special Care Dentistry Specialists (10), Mexico lacks a formal specialty in this field, thus, the care of medically complex dental patients is often handled by general dentists or specialists without specific training in this area, highlighting an urgent need for the creation of a structured specialization and the collaboration of the specialists in OM.

Multidisciplinary management was a key feature of this study, with approximately one-third of patients requiring referral to other healthcare services, a finding consistent with previous works (8,14,16). Recent studies emphasize that integrating OM with other medical and dental specialties, and even at undergraduate level, significantly improves clinical outcomes, particularly in cases involving potentially malignant disorders, oral manifestations of systemic diseases, and chronic orofacial pain (28-30). Such interdisciplinary collaboration optimizes diagnostic accuracy and treatment efficacy, fostering strong professional networks between OM and specialties such as dermatology, rheumatology, oncology, and internal medicine, thereby enhancing comprehensive patient care.

In conclusion, this study provides crucial insights into the prevalence of various oral disorders managed by OM specialists in Mexico City, serving as a foundation for defining priority areas in patient care. The findings emphasize the need to clearly define OM curricula and training programs at both undergraduate and postgraduate levels, as well as the importance of strengthening continuing education for healthcare professionals. The diverse range of pathologies treated, the complexity of therapeutic approaches, and the frequent need for multidisciplinary management highlight the essential role of OM specialists within the healthcare system. These results can serve as a basis for developing educational strategies, optimizing referral pathways, and improving the overall quality of OM care, considering the specific needs of local populations. Future research should expand this analysis to other regions in Mexico and Latin America, allowing for a more comprehensive understanding of OM practice nationwide and facilitating the development of standardized clinical and educational guidelines.

## Figures and Tables

**Table 1:** Sociodemographic and clinical characteristics of 1,270 referred patients by type of institution.

		University	Hospital	Private
		(n = 310)	(n = 306)	(n = 654)
		n ( % )	n ( % )	n ( % )
Sex	Men	96 (31.0)	77 (25.2)	187 (28.6)
	Women	2 (69.0)	229 (74.8)	467 (71.4)
Median age (Q1-Q3) (years)		50 (31-65)	56 (43-66)	60 (48-70)
Comorbidities	1	92 (49.2)	82 (35.4)	183 (34.5)
	>1	95 (50.8)	150 (64.6)	347 (65.5)
Drug use	1	43 (28.3)	46 (23.6)	92 (20.3)
	>1	109 (71.7)	149 (76.4)	362 (79.7)
Total consultations (median)		2	4	2
Seen by other professionals		103 (33.2)	110 (35.9)	382 (58.4)

**Table 2:** Primary reasons for referring patients to oral medicine services across clinical settings.

.	University	Hospital	Private
	(n = 310)	(n = 306)	(n = 654)
	n ( % )	n ( % )	n ( % )
Tumour	148 (47.7)	79 (25.8)	60 ( 9.2)
Burning mouth	28 ( 9.0)	44 (14.4)	176 (26.9)
Oral ulcers/sores	30 ( 9.7)	51 (16.7)	118 (18.0)
Dry mouth	9 ( 2.9)	47 (15.3)	60 ( 9.2)
Orofacial pain	24 ( 7.7)	21 ( 6.9)	47 ( 7.2)
White lesion	13 ( 4.2)	7 ( 2.3)	37 ( 5.7)
Red lesion	13 ( 4.2)	5 ( 1.6)	34 ( 5.2)
Pigmented lesion	18 ( 5.8)	12 ( 3.9)	15 ( 2.3)
Lip lesions	7 ( 2.3)	12 ( 3.9)	17 ( 2.6)
Assessment of systemic condition	2 ( 0.6)	14 ( 4.6)	20 ( 3.1)
Chemosensory/gustative alteration	2 ( 0.6)	2 ( 0.7)	25 ( 3.8)
Others	16 ( 5.2)	12 ( 3.9)	45 ( 6.9)

Others: gingivitis/periodontitis, radiographic finding, infection, oncological assessment, glossitis, hemorrhagic lesion, general review, dermatosis, dental treatment, facial edema, pharyngitis.

**Table 3:** Diagnostic and therapeutic activities in Oral Medicine services by type of institution.

		University	Hospital	Private
		(n = 310)	(n = 306)	(n = 654)
		n ( % )	n ( % )	n ( % )
Diagnostic tests	Biopsy	148 (47.7)	134 (43.8)	87 (13.3)
	Blood test	150 (48.4)	140 (45.8)	56 ( 8.6)
	Imaging studies	65 (21.0)	32 (10.5)	35 ( 5.4)
	Sialometry	11 ( 3.5)	82 (26.8)	4 ( 0.6)
	Exfoliative cytology	34 (11.0)	24 ( 7.8)	28 ( 4.3)
	Salivary gland flow stimulation	5 ( 1.6)	26 ( 8.5)	54 ( 8.3)
	Fluorescent light test	0 ( 0.0)	9 ( 2.9)	47 ( 7.2)
	Consultation with other medical services	3 ( 1.0)	42 (13.7)	2 ( 0.3)
	Other*	22 ( 7.1)	8 ( 2.6)	12 ( 1.8)
Treatment procedures	Drug prescription	153 (49.4)	234 (76.5)	515 (78.7)
	Therapeutic substances**	50 (16.1)	142 (46.4)	315 (48.2)
	Self-care recommendations	57 (18.4)	100 (32.7)	86 (13.2)
	Dental appliances (oclusal)	19 ( 6.1)	23 ( 7.5)	15 ( 2.3)
	Traumatic factors elimination	31 (10.0)	1 ( 0.3)	3 ( 0.5)
	Physiotherapy	3 ( 1.0)	9 ( 2.9)	8 ( 1.2)
	Surgical treatment	7 ( 2.3)	0 ( 0.0)	1 ( 0.2)
	Other***	7 ( 2.3)	0 ( 0.0)	0 ( 0.0)

*Diascopy, Nikolsky sign, supravital staining, rubbing test for pigmented lesions, vitality test, microbiological culture, allergy test, Valsalva test; **Nutritional supplements, antiseptics, dental additives, saliva substitutes, lip moisturizers, healing promoters; ***Extraction, radiographic control, dental prophylaxis.

**Table 4:** Clinical diagnoses of patients attending oral medicine services by type of institution.

.	University	Hospital	Private
	(n = 310)	(n = 306)	(n = 654)
	n ( % )	n ( % )	n ( % )
Non-odontogenic infections	81 (26.1)	129 (42.2)	125 (19.1)
Other benign oral conditions	58 (18.7)	66 (21.6)	135 (20.6)
Ulcerative and vesiculobullous lesions	31 (10.0)	69 (22.5)	93 (14.2)
Oral potentially malignant disorders	19 ( 6.1)	26 ( 8.5)	143 (21.9)
Reactive and benign neoplasm	75 (24.2)	41 (13.4)	35 ( 5.4)
Non-neoplastic salivary gland disorders	26 ( 8.4)	71 (23.2)	52 ( 8.0)
Conditions due to pharmacological effects	7 ( 2.3)	33 (10.8)	94 (14.4)
Orofacial pain and chemosensory disorders	11 ( 3.5)	20 ( 6.5)	86 (13.2)
Normal variations	23 ( 7.4)	27 ( 8.8)	43 ( 6.6)
Allergic and immunological disorders	6 ( 1.9)	17 ( 5.6)	44 ( 6.7)
Benign non-neoplastic pigmented lesions	15 ( 4.8)	15 ( 4.9)	17 ( 2.6)
Malignant neoplasms	16 ( 5.2)	5 ( 1.6)	24 ( 3.7)
Nutrimental deficiencies	2 ( 0.6)	9 ( 2.9)	31 ( 4.7)
Odontogenic infections	4 ( 1.3)	9 ( 2.9)	8 ( 1.2)
Dental and periodontal diseases	6 ( 1.9)	4 ( 1.3)	8 ( 1.2)
Odontogenic and non-odontogenic cysts	4 ( 1.3)	6 ( 2.0)	2 ( 0.3)
Syndromic disorders	0 ( 0.0)	3 ( 1.0)	3 ( 0.5)
Oral manifestations of systemic diseases	1 ( 0.3)	0 ( 0.0)	2 ( 0.3)
Others**	10 ( 3.2)	6 ( 2.0)	9 ( 1.4)
None	9 ( 2.9)	1 ( 0.3)	8 ( 1.2)

*483 patients had >1 lesion: 483 patients; **Radiographic finding, perioral seborrheic dermatitis, submucosal herniation, tonsillar hypertrophy, lymphadenitis, osteosclerosis, inflammatory sinonasal polyp, foreign body inflammatory reaction, tonsillolithiasis.
